# A comprehensive characterisation of the fibre composition and properties of a limb (*Flexor digitorum superficialis, membri thoraci*) and a trunk (*Psoas major*) muscle in cattle

**DOI:** 10.1186/1471-2121-9-67

**Published:** 2008-12-15

**Authors:** Natalia Moreno-Sánchez, Clara Díaz, María J Carabaño, Julia Rueda, José-Luis L Rivero

**Affiliations:** 1 Departamento de Mejora Genética Animal, INIA (Instituto Nacional de Investigación y Tecnología Agraria y Alimentaria), Ctra. A Coruña km 7.2, 28040 Madrid, Spain; 2 Departamento de Genética, Facultad de Biología, Universidad Complutense de Madrid, José Antonio Novais 2, 28040 Madrid, Spain; 3 Laboratorio de Biopatología Muscular, Departamento de Anatomía y Anatomía Patológica Comparadas, Facultad de Veterinaria, Universidad de Córdoba, Ctra. Madrid-Cádiz km 396, 14071 Córdoba, Spain

## Abstract

**Background:**

The fibre type attributes and the relationships among their properties play an important role in the differences in muscle capabilities and features. Comprehensive characterisation of the skeletal muscles should study the degree of association between them and their involvement in muscle functionality. The purposes of the present study were to characterise the fibre type composition of a trunk (*Psoas major*, PM) and a limb (*Flexor digitorum, membri thoraci*, FD) muscle in the bovine species and to study the degree of coordination among contractile, metabolic and histological properties of fibre types. Immunohistochemical, histochemical and histological techniques were used.

**Results:**

The fibre type composition was delineated immunohistochemically in calf muscle samples, identifying three pure (I, IIA, and IIX) and two hybrid type fibres (I+IIA, and IIAX). Most of the fibres in FD were types I and IIA, while pure IIX were absent. All fibre types were found in PM, the IIX type being the most frequent. Compared to other species, small populations of hybrid fibres were detected. The five fibre types, previously identified, were ascribed to three different acid and alkaline mATPase activity patterns. Type I fibres had the highest oxidative capacity and the lowest glycolytic capacity. The reverse was true for the IIX fibres, whereas the type IIA fibres showed intermediate properties. Regarding the histological properties, type I fibres tended to be more capillarised than the II types. Correlations among contractile, metabolic and histological features on individual fibres were significantly different from zero (r values varied between -0.31 and 0.78). Hybrid fibre values were positioned between their corresponding pure types, and their positions were different regarding their metabolic and contractile properties.

**Conclusion:**

Coordination among the contractile, metabolic and histological properties of fibres has been observed. However, the magnitude of the correlation among them is always below 0.8, suggesting that the properties of muscles are not fully explained by the fibre composition. These results support the concept that, to some extent, muscle plasticity can be explained by the fibre type composition, and by the properties derived from their metabolic and histological profiles.

## Background

Myofibres are the functional units of individual skeletal muscles. Such muscles consist of a heterogeneous population of fibres, differing in their molecular, structural, contractile and metabolic features, which contribute to a wide variety of functional capabilities. Up to now, the different isoforms of the myosin heavy chain (MyHC) protein have seemed to be the best markers to characterise muscle fibre type diversity [1]. Traditionally, studies in the field of muscle research have relied on a histochemical classification based on the staining for acid or alkaline stabilities of the myofibrillar ATPase (mATPase) activity [2]. The alternative, of immunohistochemistry with specific poly/monoclonal antibodies, was identified as a much more objective method for the accurate identification of muscle fibre types according to the MyHC isoform they express. This is due to the ability of such a method to identify hybrid fibres [3], which show the coexistence of several MyHC isoforms. This coexistence in a single fibre is detected by monoclonal antibodies (MAbs) against the different MyHC isoforms; the predominant isoforms being responsible for the fibre's functional properties, such as the speed of contraction and the fatigue resistance [4].

Eight MyHC isoforms have been identified in adult bovine striated muscles, including cardiac, developmental, adult and extraocular isoforms [5,6]. All eight isoforms are co-expressed in extrinsic eye muscles, three (I, IIA and IIX) in limb and trunk muscles and two (I and α) in masseter. The expressions of IIB and Eo are restricted to extraocular muscles, and developmental isoforms are only found in specialised muscles in the larynx and in the eye. The expression of MyHC IIB represents a controversial issue in cattle. While Chikuni and co-workers [7,8] concluded that a functional gene coding for MyHC IIB was not present in the genome of all ungulates they examined, Toniolo and co-workers [9] proposed that the expression of IIB and Eo in extraocular muscles could be related to an embryological origin or to their specialised contractile requirements. They also suggested that fibres expressing MyHC-α are particularly suited for the bovine diet and the chewing action. Bovine trunk and limb muscles consist of a population of type I or slow fibres, and two fast isoforms, or type II (IIA and IIX) [3] similarly to humans [10], carnivores [11], small ruminants [12] and horses [13], but with a lower proportion of hybrid fibres. The sequencing and expression of the genes coding MyHC proteins have previously been reported in different bovine muscles [5,6]. Fibre type composition from similar skeletal muscles seems to differ among species [3,9,14] and breeds within the same species [15,16], somehow reflecting a process of adaptation to the animals' functionality (the functions that the animal carries out, such as grazing, ruminating, movement, etc.) and/or the result of selection towards a breed specialisation [17].

Muscle specialisation is the result of the coordinated expression of contractile and metabolic proteins together with the histological features that characterise the fibres. Studies characterising and relating contractile, metabolic and morphological attributes of muscle fibres have been performed in several species [11,18]. These studies show that cellular expression of the various MyHC isoforms is coordinated with the metabolism (oxidative or glycolytic), the activity of myofibrillar adenosine triphosphatase (mATPase), and some morphological features. However, to what extent the pattern of coordination is maintained across species is unknown and, therefore, extrapolation of results across species should be performed cautiously. Muscle plasticity is an intense area of research. Plasticity is mainly characterised by changes in the expression of tissue specific isoforms and the ability to undergo adaptive metabolic processes [19]. These mechanisms of muscle plasticity may somehow affect the pattern of coordination within and across species. From this perspective, accomplishing comprehensive studies to characterise the coordination of properties responsible for muscle capabilities appears to be of interest. To the best of our knowledge, this type of study has not yet been performed in cattle on a fibre to fibre approach. The understanding of such interrelationships is important to ascertain aspects related to beef quality differences as well as to muscle diseases.

In this study we characterised fibre type composition and features in two muscles, *M. psoas major *(PM), and *M. flexor digitorum superficialis, membri thoraci *(FD), which were representative of two meat cuts. These two cuts clearly differ in their meat quality attributes. Among other properties, PM muscles have more intramuscular fat content and are more tender than FD [20].

The purpose of the present study is twofold: firstly, to characterise the fibre type composition of these two bovine skeletal muscles by using a combination of inmunohistochemical and histochemical techniques which allows us to identify the MyHC isoforms they exhibit; Secondly, to examine accurately the interrelationships of relevant contractile, metabolic and histological properties of bovine muscle fibre types on a fibre-to-fibre basis.

## Methods

### Muscle samples

Male calves of the Avileña-Negra Ibérica breed were sampled for the experiment. Animals were fattened under the same diet and location conditions, and slaughtered when they were fit for commercial requirements, which is to say at a weight of about 500 kg, normally achieved at around 450 days. Samples of approximately 1 × 1 × 0.5 cm^3 ^were removed from the superficial layer of the PM and FD muscles from each calf. These two muscles were chosen because of their different anatomical locations and potentially distinctive functional roles in motion. While PM is a dynamic muscle that causes the propulsion of the leg, and is involved in activities of high energy cost, the FD is a postural muscle that is involved in long activities of low energy cost. Moreover, these two muscles are part of two specific meat cuts which markedly differ with respect to relevant meat quality traits [20]. Two samples (one per muscle) of each of five calves (n = 10, 2 per animal) were collected after slaughter.

Muscle samples were placed on a suitable piece of cork, allowing their relaxation for a few minutes, covered with OCT™ Compound (Tissue-tek^®^, Sakura Finetek) and then frozen in isopentane chilled in liquid nitrogen. They were stored at -80°C until analysis.

Serial cross sections were cut in a cryostat at -20°C, and placed on labelled and coated glass slides for immunohistochemistry, histochemistry and histology. Two consecutive sections from each muscle and individual were used for each of the procedures described below.

### Immunohistochemistry. Fibre typing

Four monoclonal antibodies (MAbs), BAF8, SC71, BF35, and S58H2, whose specificities for the MyHC isoforms have been previously demonstrated in mammals, were used [3,21,22]. Their source and specificities are in the Acknowledgement section and Table 1, respectively, and also described elsewhere [3,21]. Serial sections of 10 μm from both muscles were pre-incubated in a blocking solution of stock goat serum for each MAb, and then incubated overnight at 4°C with the primary MAb. The working dilutions were 1/300 in PBS for BAF8, SC71 and BF35, and 1/50 in PBS for S58H2. An additional section was incubated without specific primary MAb and used as blank tissue to demonstrate the non-specific reactivity and control the background staining.

**Table 1:** Specificity of the monoclonal antibodies used in the immunohistochemistry

	**Cattle skeletal muscle fibre types**				
**Monoclonal antibodies**	**I**	**I+IIA**	**IIA**	**IIAX**	**IIX**

BAF8	+	+	-	-	-
SC71	-	+	+	+	+
BF35	+	+	+	+	-
S58H2	+	+	-	+	+

Positive (+) or negative (-) reactions of specific myosin heavy chain isoform or fibre type to each monoclonal antibody.

After incubation, the sections were washed and incubated with the secondary antibody (biotinylated goat anti-mouse IgG; code n° E0433; Dako) for 30 minutes. Sections were washed again and reacted for 1 hour in the dark with ABC reagent. The immunocomplexes were visualised by incubating the sections for 2–3 minutes in a diaminobenzidine solution. After being stained, TRIS solution (pH 7.6) and tap water were applied to the slides to stop the progress of the staining. The slides were dehydrated in an ethanol series, cleared in xylol and cover-slipped with DPX resin for microscopy (BDH Lab Supplies, Poole, England).

The fibres were classified according to their MyHC content by means of visual examination of the immunostained serial sections. After the image analysis of consecutive frames (each of the four sections stained with a particular anti-MyHC MAb), the reactivity of each fibre was judged as positive or negative by comparing the staining intensity with that of the neighbouring fibres. Five fibre types were characterised as I, I+IIA, IIA, IIAX or IIX according to the pattern of immunoreactivity shown in Table 1.

### Histochemistry: Myofibrillar ATPase activity and metabolic properties

Additional 10 μm serial sections were stained for mATPase activity after acid and alkaline preincubations by using a modification of the Brooke and Kaiser (1970) method [23]. The optimum pH for both mATPase denaturation protocols was carefully searched in each muscle, in order to visually distinguish at least two or three intensity levels of staining. For this purpose, serial sections were stained using a range of pH values, from 4.25 to 4.55 in increments of 0.05 for the acid preincubation, and from 10.25 to 10.50 for the alkaline one, likewise in increments of 0.05. The optimum values were 4.42 and 10.35 for the acid and the alkaline preincubation respectively. This mATPase histochemical approach was pursued to characterise the acid and alkaline stabilities of the mATPase activity in bovine skeletal muscle fibre types, which had been classified according to their differential MyHC content. These activities are not only species-specific [24] but they can also vary greatly within the same species according to the mATPase histochemical method employed [25].

The histochemical activity of succinate dehydrogenase (SDH) enzyme was used as a marker of the oxidative capacity of muscle fibres. Staining was carried out on 10 μm thick sections according to the histochemical procedure previously described [26], except for the optimum incubation time which was 10 minutes. The histochemical activity of glycerol-3-phosphate-dehydrogenase (GPDH) enzyme was used as an indirect marker of the glycolitic capacity of muscle fibres. Staining was carried out on 14 μm thick sections according to Martin et al., [27], except for the incubation time which was increased to 45 minutes. The linearity of the quantitative histochemical reactions (SDH and GPDH) in relation to the incubation time has been previously verified in a number of mammalian species [11,18]. Optical density (OD) was measured in the stained sections, as it has been demonstrated that a high degree of analytical precision can be achieved by measuring the OD of the fibres on histochemical sections [26,27].

### Histology

Additional 14 μm thick serial sections were stained according to a standardised periodic-acid-Schiff (PAS) technique for selective staining of glycogen in individual fibres, using a 1% acid solution for 5 min at 37°C. Other sections of 14 μm thickness were incubated for 60 min at 37°C in a 2.2% α-amylase (Sigma Chemical Co., St Louis, MO, USA. Product No. A-2771) solution and then were stained with the same PAS protocol [28]. These sections were used both to visualise capillaries and to measure the cross sectional area (CSA) of individual fibres. Additional 10 μm thick sections were stained with haematoxylin and eosin to determine the number of nuclei.

The absolute values of both nuclei and capillaries for each fibre were converted to relative values by dividing them by the CSA of the corresponding fibre. This staining did not distinguish myonuclei from other nucleus types (intrafibre nuclei, and nuclei of the satellite cells and capillaries). The number of nuclei was obtained by counting all the nuclei around each individual fibre.

### Image analysis and morphometry

Sections were visualised and analysed by a Leica DMLS microscope (Leica Microsistemas, Barcelona, Spain), a Leica high-resolution colour charge-coupled device camera (Leica Microsistemas, Barcelona, Spain), an eight-bit Matrox Meteor frame-grabber (Matrox Electronic Systems, Barcelona, Spain) and the Scion Image (ScnImage) software (Scion Corporation, Maryland, USA, available at ).

All sections were carefully surveyed to find regions which were free of artefacts, and ten regions (five per muscle) were taken for analyses. These regions contained between 50 and 128 fibres (mean, 81 fibres). A minimum of 15 fibres of each type were present in each region, except hybrid I+IIA and IIAX types, due to their low frequency. Fibres in each area were individually identified, and a fibre mask was manually drawn along the edge of each fibre, for the inmunohistochemical, histochemical and histological assays. The CSA and OD were determined for each fibre. The CSA was measured in the α-amylase PAS stained sections, as this staining does not have a negative impact on fibre size. The numbers of capillaries and nuclei around each numbered fibre were obtained from the α-amylase-PAS and haematoxylin-eosin staining techniques, respectively. They were expressed in both absolute and relative terms (as the number of capillaries or nuclei per 1,000 μm^2 ^of the fibre CSA).

A total number of 814 individual muscle fibres, 455 in FD and 319 in PM, could be fully characterised in all the sections of the ten samples (5 specimens by 2 muscles).

Substantial variations in ODs were detected between different muscle specimens for all immunohistochemical and histochemical stainings. Accordingly, the OD of each fibre was normalised by means of the *Z scores *within each muscle based on the following algorithm:

$$Z=\frac{X_{i}-\bar{x}}{s}$$

where *X*_*i *_is an individual OD measure, $\bar{x}$ is the mean for all fibres within a group (fibre population, digitalised image and individual muscle sample), and *s *is the standard deviation [29]. Standardisation to *Z *score was carried out separately for each fibre type within a digitalised image, for each digitalised image within each individual muscle sample, and for each individual muscle sample within each skeletal muscle. The resultant positive and negative *Z *values were then expressed on a scale ranging from 0 to 1.

### Statistical analysis

Statistics and charts were obtained by the Statistical Advisor software (StatSoft, Inc. 2001. STATISTICA data analysis software system, version 6. ). Descriptive statistics were used to derive means, SE and 0.95 confidence intervals for all variables. Statistical analyses of each dependent variable were carried out using a two-way analysis of variance (ANOVA) including the effects of fibre type and muscle, and the interaction between them. In the presence of a significant F ratio, post hoc comparisons of means were provided by a Fisher's least significance difference test. Statistical significance was accepted at p < 0.05. In general, variations attributable to the muscle of origin were low, although not totally absent, for the immunohistochemical and histochemical features of the fibre types, but they were significant for the morphological features. Accordingly, data for immunohistochemical and histochemical variables of myofibres are shown as pooled means of the total number of analysed fibres, in the two muscles, and in the five animals. However, data concerning CSA, capillaries and the total nuclei of the fibre types are presented separately for each muscle. Pearson's coefficients of correlations were also obtained for specific fibre types within and between muscles, in order to estimate the degree of interrelationships among different muscle fibre type characteristics.

The method of canonical discriminant analysis was applied for the study of the relationships between the different techniques. This method is a dimension-reduction technique related to principal component analysis and canonical correlation, in which linear combinations of the quantitative variables are found which provide maximal separation between the classes or groups, the five fibre types in our case. The procedure computes squared Mahalanobis distances between class means. This analysis describes collectively the relationship among all variables, and compares individual muscle fibres by simultaneously considering all the quantified variables. Plotting pairs of canonical variables for all the observations, fibres in our case, provided an overall view of the coordination of contractile, metabolic and morphological features of the fibre types.

## Results

### Immunohistochemistry. Fibre typing

**Figure 1:**
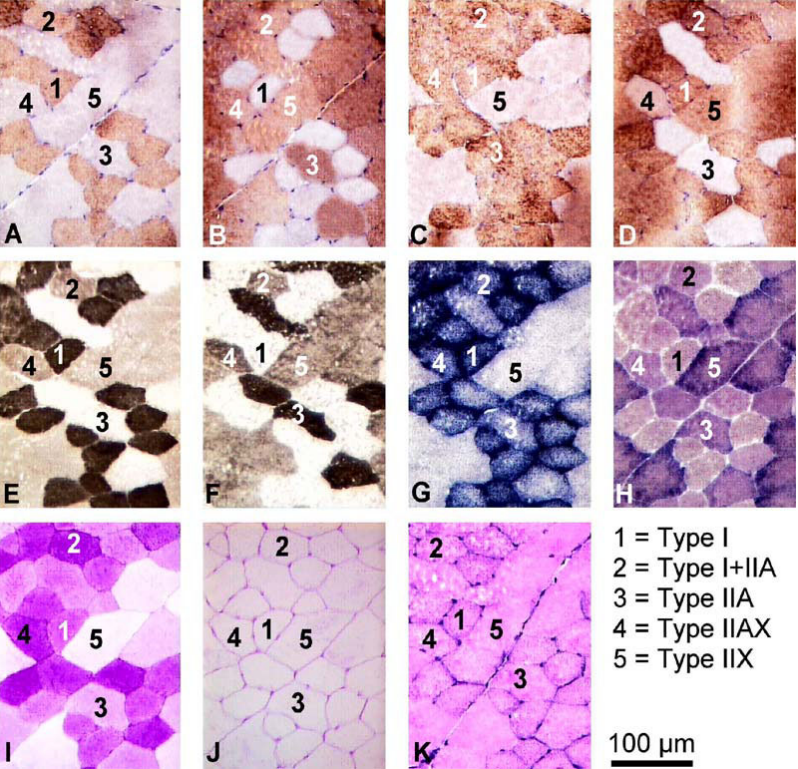
**Serial sections of the PM muscle stained for immunohistochemistry, enzyme histochemistry and histology**. A-D: Sections were stained with a battery of MAbs against specific MyHC isoforms: BAF8 anti MyHC I (A), SC71 anti MyHC IIA and IIX (B), BF35 anti MyHC I and IIA (C), and S58H2 anti MyHC I and IIX (D). E-F: Sections assayed for mATPase activity after acid (pH 4.42, E) and alkaline (pH 10.35, F) preincubations. G-I: Sections assayed for SDH (G), GPDH (H), and PAS for selective staining of glycogen (I). J-K: Histological staining with PAS after digestion with α-amylase to reveal capillaries (J) and haematoxylin-eosin to show total nuclei (K).

The fibre types were identified by visual inspection of sections stained with the anti-MyHCs MAbs. Three of them were pure fibre types expressing a unique MyHC isoform, either I, IIA or IIX, and two others were hybrid types co-expressing two MyHC isoforms, I plus IIA (I+IIA), and IIA plus IIX (IIAX). Type I fibres reacted with all MAbs except SC71 (e.g. fibre 1 in Figure 1A–D). Type IIA reacted with MAbs SC71 and BF35 but not with the remaining MAbs (e.g. fibre 3 in Figure 1A–D). Type IIX fibres were negative for MAbs BAF8 and BF35, and positive for S58H2 and SC71 MAbs (e.g. fibre 5 in Figure 1A–D). Hybrid I+IIA fibres reacted with all four MAbs (e.g. fibre 2 in Figure 1A–D), while IIAX fibres were labelled with all MAbs, except BAF8 (e.g. fibre 4 in Figure 1A–D). The fibre immunostaining with the specific anti-MyHC MAbs showed a wide range of reactions, not just positive or negative. In the hybrid types, the staining showed a continuous variation, possibly due to the differential contents of the MyHCs they are co-expressing.

Table 2 shows fibre composition for the FD and the PM muscles. On average, the FD muscle was mainly composed of type I and IIA fibres. The remaining fibres were the hybrid types, I+IIA and IIAX, but pure IIX fibres were not found in any of the FD muscle specimens examined. PM muscle had on average a balanced proportion among the three main pure fibre types, and a reduced number of hybrid fibres. The amount of hybrid fibres changed according to the muscle, there being more I+IIA in FD than in PM, while the opposite was true for the IIAX type.

**Table 2:** Different fibre types in each of the two studied muscles

		**MyHC Muscle Fibre Types**					
**Muscle**		**I**	**I+IIA**	**IIA**	**IIAX**	**IIX**	**Total**

FD	n	194	23	131	11	0	359
	n/total	0.54	0.06	0.36	0.03	0.00	

PM	n	132	20	94	48	161	455
	n/total	0.29	0.04	0.21	0.10	0.35	

Number (n) and proportion (n/total)^1 ^of the different fibre types pooled from five animals in each of the two studied muscles.

^1^Percentages of the different fibre types were not calculated because data came from five pooled animals.

**Figure 2:**
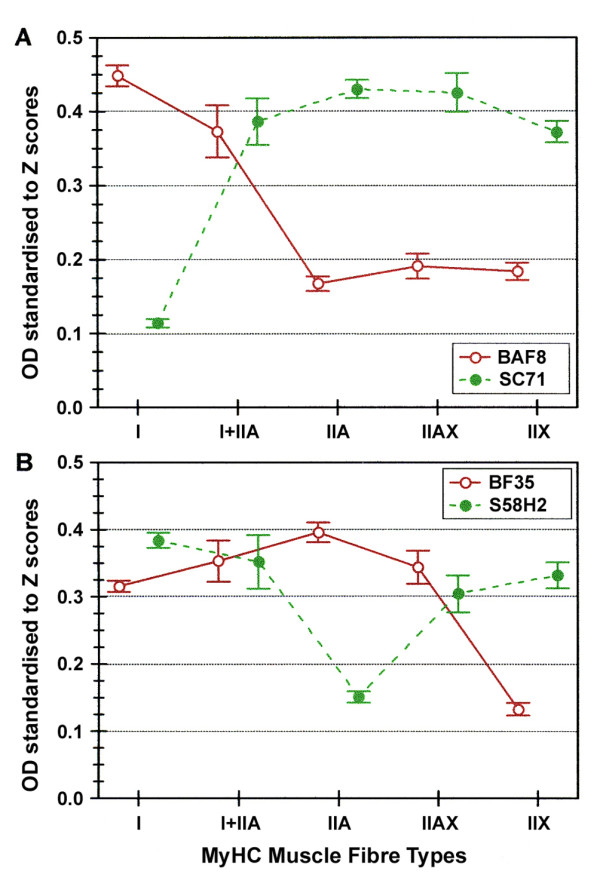
**Normalised mean OD of MyHC-based fibre types stained with four anti-MyHC Mabs**. BAF8 (anti MyHC I) and SC71 (anti MyHC IIA and IIX) (A), BF35 (anti MyHC I and IIA) and S58H2 (anti MyHC I and IIX) (B). Values are pooled means of the 814 fibres analysed in the two skeletal muscles (PM and FD). Vertical bars represent the 0.95 confidence intervals.

Quantitative differences in the immunostaining of MyHC observed among fibre types were significant for all MAbs. The BAF8 MAb allowed the discrimination amongst type I and I+IIA fibres while the SC71 MAb labelled type II fibres (Figure 2A). The BF35 MAb labelled all fibre types except IIX fibres, while S58H2 stained positively all fibre types but IIA (Figure 2B).

### Myofibrillar ATPase activity

Based on the visual examination of acid and alkaline mATPase reactions, bovine skeletal muscle fibre types could be assigned to three main categories, corresponding to the three main staining intensities (Figure 1E–F). Type I fibres were acid-stable and alkaline-labile (see fibre 1 in Figure 1E–F) whereas type IIA ones were acid-labile and alkaline-stable (see fibre 3 in Figure 1E–F), and type IIX ones were partially acid- and alkaline-stable (see fibre 5 in Figure 1E–F). Hybrid I+IIA and IIAX fibre types showed mATPase activities between their respective pure MyHC fibre types.

**Figure 3:**
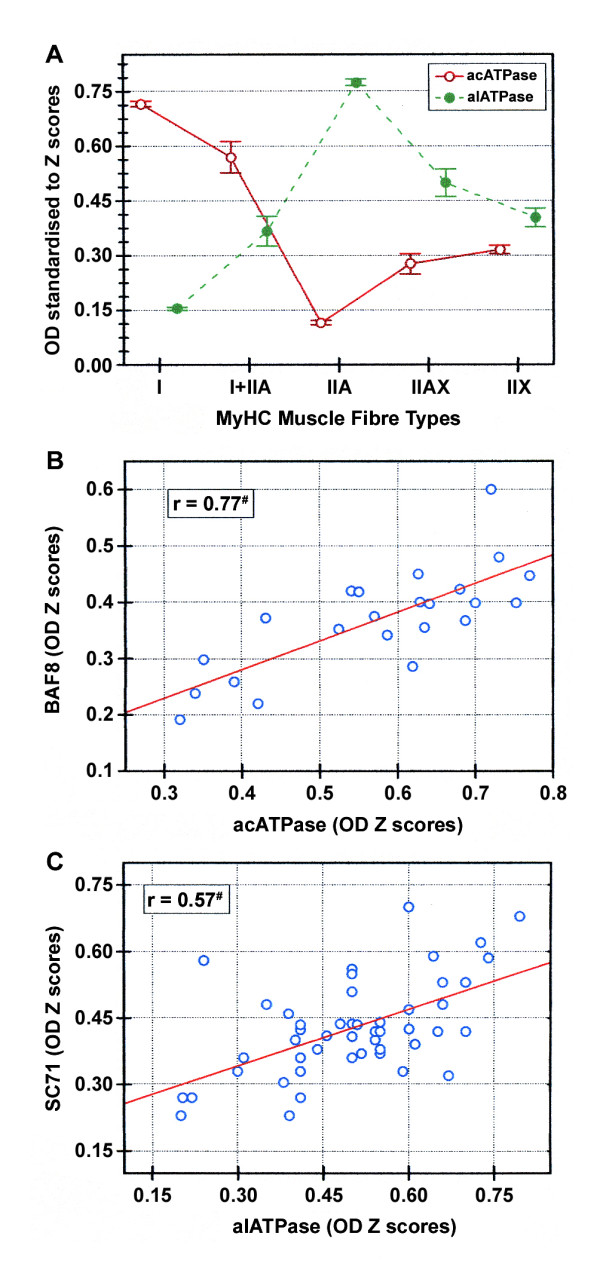
**Normalised mean OD of mATPase activities and their relationships to the fibre types**. A: Mean OD standardised to Z scores of mATPase activity after acid (pH 4.42, acATPase) and alkaline (pH 10.35, alATPase) preincubations of the MyHC-based fibre types. Values are pooled means of the 814 fibres analysed in the two skeletal muscles (PM and FD). Vertical bars represent the 0.95 confidence intervals. B, C: Fibre-to-fibre relationship between OD of the immunostaining and mATPase activities in different subsets of hybrid fibres. Relationship between the BAF8 MAb (anti MyHC I) and acATPase staining of all I+IIA hybrid fibres analysed in the FD (n = 23 fibres) (B). Relationship between the SC71 MAb (anti MyHC IIA and IIX) and alATPase staining of all IIAX hybrid fibres identified in the PM (n = 48 fibres) (C). r: Pearson coefficient of correlation; ^#^: p < 0.001 level of significance.

Quantitative differences in the staining of both mATPase assays, related to the previously established fibre types, are shown in Figure 3A. Significant differences among fibre types were detected for both acid and alkaline mATPase activities. Pure fibre types could be clearly distinguished on serial sections stained with these two techniques while hybrid fibres overlapped with their respective pure types. Hybrid I+IIA fibres were closer to I than to IIA, and IIAX were closer to IIX than to IIA (Figure 3A).

The degree of correlation between the mATPase histochemistry and the MyHC expressed in a given fibre can be examined by plotting OD values of either acid or alkaline mATPase against specific anti-MyHC MAbs. As the data did not follow a monotonically increasing function across classes, the relationships between the mATPase stainings and the different MyHC types could only be calculated in the hybrid fibre populations (Figure 3B–C). The hybrid fibres showed a continuous variation in the staining intensity as a function of their different MyHC contents. A positive and significant correlation was observed between the acid mATPase and the BAF8 MAb stainings of the hybrid I+IIA fibres in the FD muscle (Figure 3B). This correlation clearly indicated an increase of the acid mATPase stability of I+IIA fibres as the MyHC proportion changed from mainly type I to mainly type IIA. Similarly, a significant positive correlation was observed between the alkaline mATPase and the SC71 MAb stainings of the hybrid IIAX fibres in the PM muscle (Figure 3C). Once again, alkaline mATPase in those fibres varied as the MyHC proportion changed from mainly IIA to mainly IIX.

Taking into account the correlations between OD values of specific anti-MyHC MAbs and OD values of mATPase, hybrid fibres appeared to have a continuous and linear transition between their corresponding pure types. Therefore, hybrid fibres represented a heterogeneous population between pure fibres, in which the association between contractile, metabolic and histological attributes can be fruitfully investigated.

### Metabolic properties

**Figure 4:**
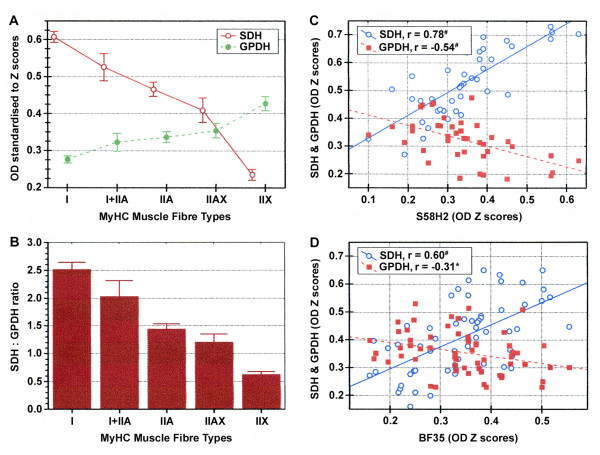
**Normalized mean OD of SDH and GPDH activities and their relationships to the fibre types**. A, B: OD standardised to Z scores of SDH and GPDH activities of the MyHC-based fibre types. Mean OD for SDH and GPDH (A). SDH:GPDH ratio (B). Values are pooled means of the 814 fibres analysed in the two skeletal muscles (PM and FD). Vertical bars represent the 0.95 confidence intervals. C, D: Fibre-to-fibre relationships between OD of the immunostaining and SDH and GPDH histochemical activities in various subsets of hybrid fibres. Relationship between the S58H2 MAb (anti MyHC I and IIX) and both SDH and GPDH staining of all I+IIA hybrid fibres identified in the present study (n = 42 fibres) (C). Relationship between the BF35 MAb (anti MyHC I and IIA) and both SDH and GPDH staining of all IIAX hybrid fibres identified in the study (n = 59 fibres) (D). r: Pearson coefficient of correlation; ^#^, *: p < 0.001 and p < 0.05 levels of significance, respectively.

The visual examination of SDH and GPDH histochemical reactions revealed a continuous variation in the staining intensities of all fibre types (Figure 1G–1H). Significant differences were detected among fibre types for SDH and GPDH histochemical activities (Figure 4). On average, SDH mean activities tended to decrease significantly from type I to type IIX fibres, whereas GPDH mean activities showed the reverse tendency (Figure 4A). Hybrid I+IIA fibres were slightly closer to IIA than to I, and IIAX were closer to IIA than to IIX (Figure 4A). Therefore, the ratio SDH:GPDH, which is commonly used as an indicator of the rate between oxidative and glycolytic metabolisms of myofibres, decreased consistently, as shown in Figure 4B. SDH activity was higher than GPDH activity in all fibre types but IIX, which showed higher GPDH than SDH activities. A negative correlation (r = -0.49) was found between SDH and GPDH activities.

In order to ascertain the degree of coordination between metabolic enzymes and contractile proteins, SDH and GPDH reactions were plotted against anti-MyHC MAbs stainings of hybrid fibres I+IIA (Figure 4C) and IIAX (Figure 4D). As the data did not follow a monotonically increasing function across classes, these relationships were only calculated for the hybrid fibre populations. In both cases, the magnitude of the correlations (Figures 4C and 4D) between SDH activity and MyHC isoforms was larger than that with GPDH activity. As expected, hybrid I+IIA fibres which contained more IIA than I MyHC, showed lower values of SDH and larger values of GPDH. Similarly, hybrid IIAX fibres containing more IIX than IIA MyHC had lower SDH and higher GPDH values than fibres containing more IIA than IIX.

The PAS staining (Figure 1I) showed that the glycogen content of each individual fibre decreased significantly from I to IIX fibre types, contrary to other results [18]. More severe stress-induced glycogen depletion was observed in all fibre types of the FD muscle than in the corresponding fibre types of the PM muscle.

### Histology

The staining for CSA and nuclei determinations is shown in Figure 1J and for capillaries in Figure 1K. Mean values of CSA and absolute and relative numbers of total nuclei and capillaries are summarised in Table 3. Mean comparisons among fibre types and muscles for each of these features are also shown. In general terms, CSA were larger in FD than in PM. The absolute numbers of nuclei and capillaries tended to be larger in FD than in PM, but this pattern changed when relative values were considered. The relative values of nuclei were quite similar between muscles and the relative values of capillaries were larger in PM than in FD (Table 3).

**Table 3:** Mean ± SE of the histological measurements in each of the two studied muscles

		**MyHC Muscle Fibre Types^1^**				
**Variable**		**I**	**I+IIA**	**IIA**	**IIAX**	**IIX**

CSA (μm^2^)	FD	3246 ± 79 b	3172 ± 241 b	3962 ± 160 c	2422 ± 235 a	
	
	PM	2086 ± 102 a	1806 ± 197 a	2157 ± 136 a	2459 ± 226 a	3692 ± 154 b

Capillaries (n)	FD	4.11 ± 0.11 a	3.59 ± 0.24 a	3.98 ± 0.13 a	3.09 ± 0.37 a	
	
	PM	3.71 ± 0.14 c	3.94 ± 0.36 c	3.24 ± 0.17 bc	2.74 ± 0.27 ab	2.60 ± 0.13 a

Capillaries (n 10^3^/μm^2^)	FD	1.37 ± 0.04 b	1.20 ± 0.10 ab	1.10 ± 0.04 a	1.56 ± 0.32 ab	
	
	PM	2.36 ± 0.15 c	2.53 ± 0.30 c	1.84 ± 0.12 b	1.58 ± 0.21 b	0.90 ± 0.06 a

Nuclei (n)	FD	11.32 ± 0.35 b	9.68 ± 0.82 b	11.16 ± 0.62 b	9.70 ± 1.33 b	
	
	PM	6.22 ± 0.29 a	5.84 ± 0.65 a	5.79 ± 0.35 a	4.61 ± 0.40 a	5.71 ± 0.30 a

Nuclei (n 10^3^/μm^2^)	FD	3.82 ± 0.19 ab	3.24 ± 0.26 ab	3.07 ± 0.14 ab	3.99 ± 0.42 ab	
	
	PM	3.82 ± 0.26 b	4.01 ± 0.47 b	3.39 ± 0.19 ab	2.49 ± 0.29 a	1.93 ± 0.13 a

^1 ^See Table 2 for the number of fibre types analysed in each skeletal muscle

CSA (μm^2^), and absolute (n) and relative (n 10^3^/μm^2^) numbers of both capillaries and nuclei of the different fibre types in each of the two studied muscles are shown. Relative values are obtained by dividing the absolute values by the CSA. Post hoc comparisons of means were provided by a Fisher's least significance difference test. Means with different letters are statistically different.

Differences among fibre types became more evident when the relative values of both nuclei and capillaries were considered. The relative values of capillaries and nuclei in type I fibres tended to be higher than in the most glycolitic ones. A positive relationship (r = 0.64) was detected between relative numbers of capillaries and nuclei.

When analyzing the possible relationship between histological and metabolic properties, a negative correlation (r = -0.58) was detected between CSA and SDH activity on a fibre to fibre comparison in all PM fibres. The value of the r coefficient was influenced by the behaviour of the IIX fibres, as they showed a wide CSA range which was not associated with a large variation in SDH activity.

### Multivariate analysis

**Figure 5:**
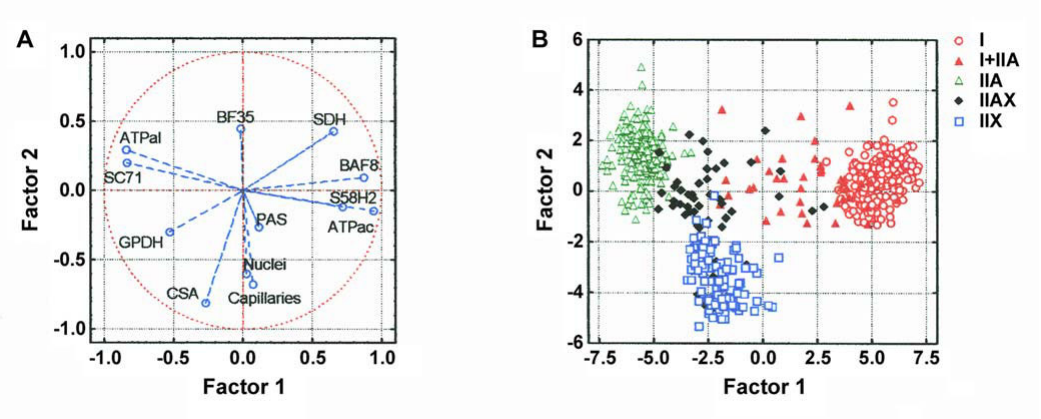
**Multivariate analysis of MyHC fibre types' features**. Spatial distribution of fibre types' features according to results of the canonical discriminant analysis (A) (see Fig. 2 to 4 for abbreviations). Spatial distribution of all fibres (n = 814) according to the first two canonical factors (B). Factor 1 indicates the position of muscle features in relation to their ability to discriminate type I (right) and type II (left) fibres. Factor 2 indicates the position of muscle features in relation to their ability to discriminate type IIA (top) and type IIX (bottom) fibres.

The dataset coming from both muscles was subjected to multivariate analyses to summarise fibre type features according to their MyHC content (Figure 5). The ability of these features to discriminate fibre types was examined by canonical discriminant analyses (Figure 5A). Almost 75% of the data variance was explained by the first two factors. The first factor 1 in Figure 5A included the most relevant variables for the distinction between type I and type II fibres: mATPase activities, and BAF8, S58H2 and SC71 MAbs, while the relevance of SDH and GPDH activities was smaller. The second factor which delineated fibre II subtypes was mostly explained by CSA and the BF35 MAb. As before, metabolic activities were also less important. These two major components allowed fibre type discrimination as shown in Figure 5B. Collectively, three major fibre type populations (I, IIA and IIX) were clearly discriminated amongst, whereas hybrid fibre types appeared in between the neighbouring major classes (Figure 5B). Thus, type I+IIA fibres were located between I and IIA, and tended to be closer to I than to IIA, while type IIAX fibres appeared between IIA and IIX, but slightly closer to IIX than to IIA fibre types. The same behaviour was observed when Mahalanobis distances among all groups of fibres were calculated (Table 4). Distances between fibre types were all statistically significant.

**Table 4:** Squared Mahalanobis Distances and F-values by discriminant analysis

**Fibre types**	**Discriminant analysis**	**MyHC Muscle Fibre Types**			
		
		**I+IIA**	**IIA**	**IIAX**	**IIX**
I	SMD	15	120	64	70

	F-values	41	983	223	411

I+IIA	SMD		65	25	36

	F-values		166	45	82

IIA	SMD			15	35

	F-values			50	188

IIAX	SMD				9

	F-values				26

Squared Mahalanobis Distances (SMD) and F-values for overall differences between fibre types by discriminant analysis on all muscle features and fibres of the two bovine skeletal muscles (n = 814 fibres). All distances among fibre types are significant p < 0.001.

## Discussion

### Immunohistochemistry. Fibre typing

The functional properties of muscles that qualify them for locomotion, postural maintenance or respiration, among others, can be elucidated from their fibre type composition. To the authors' knowledge, the FD muscle has not been previously characterised in cattle. An interesting result of the present study was the abundance of slow-twitch type I fibres and the vestigial expression of the MyHC IIX isoform in the FD, resulting in the absence of IIX pure fibres and a very low proportion of hybrid IIAX fibres (Table 2). Muscles composed mainly of type I could play a major role in maintaining posture by stabilizing the extended joints, while large muscles generating the strong power needed for propulsive force contained a high proportion of the IIX type [30], as did the PM. The fact that the IIX pure type was not found in FD indicated the specialisation of this muscle towards a greater endurance, corresponding to a muscle continuously used throughout the day for walking, standing, etc.

Similar to the results shown by Picard et al. [3] in the bovine species, the muscles studied here showed a small proportion of hybrid fibres in comparison to other mammalian species such as horses [13,18], pigs [18] or dogs [11]. The role of the hybrid fibres is not fully understood. Some authors [31] assert that they indicate the dynamic transition from one pure phenotype to the other, whereas some others [32] claim that they are stable populations that can behave differently depending on external stimuli. In this context, we speculate that a low percentage of hybrid fibres could not significantly change the behaviour of the muscle in response to a external stimuli, whereas a large population of hybrid fibres could. So, species with a low proportion of hybrid fibres (cattle) would support the "dynamic transition" hypothesis, and species having a large percentage of them (dog) the "stable population" one. As the MyHC isoform composition of a single fibre can be used as a "physiological marker", then the extent of hybridism may reveal the diversity of activity that a given muscle or species requires.

The very fast MyHC IIB was not detected in our study, in agreement with Tanabe et al., [5], Maccatrozzo et al., [6], and Toniolo et al., [9]. Although large mammalian species were initially supposed not to have the very fast MyHC IIB isoform, it has been found in some of them, such as pig *Longissimus *muscle, [33] and llama *Semitendinosus *and *Vastus lateralis *muscles [24,34]. This MyHC isoform was functionally and morphologically compatible with the MyHC IIB gene, commonly reported in eutherian species of mammals [35]. Nevertheless, this third fast MyHC isoform, compatible with the IIB isoform of small rodents, is not expressed in trunk and limb skeletal muscles of humans, carnivores, ruminants or horses [5,36-39].

The fibre type composition of muscles in meat producing breeds influences their meat quality features. A positive relationship between the percentage of type I fibres and the intramuscular fat (IMF) has been previously described for bovine skeletal muscles [15]. It is noticeable that in the present study a larger proportion of type I fibres was found in the muscle which had a lower IMF content [20]. In relation to this finding, some results in human muscles pointed out the absence of a relationship between the expression of *MHY7 *(the gene coding for MyHC I) and the expression of genes involved in adipogenesis such as *PPARα *and *PPARδ *[40]. The predominance of type II fibres has been associated with a faster post-mortem ageing rate and, therefore, to a rapid rate of tenderisation [41]. In our case, the PM showed a larger proportion of both IIA and IIX types and was perceived as a more tender muscle than the FD [20]. Both IMF and tenderness are relevant traits in the cattle production context.

In a different study, we have performed a microarray experiment to assess the differential gene expression between PM and FD muscles in male Avileña-Negra Ibérica calves [42] A bovine fat and muscle cDNA microarray [43] was used and 20 microarray slides were hybridised following a loop design that directly compared both muscles within and between individuals. *MYH7 *gene (coding for type I isoform) was more expressed in FD, which is in agreement with the higher proportion of type I fibres found in this muscle (see Table 2). Furthermore, the *MYH1 *gene (coding for type IIX isoform) was more expressed in PM, in agreement with the larger proportion of IIX fibres found in this muscle. However, the *MYH2 *gene (coding for type IIA isoform) did not show a significant difference in expression in the FD muscle, which had a higher proportion of type IIA fibres in the current experiment. This last observation has also been described in swine [14,44]. The most common explanation is that mRNA coming from different MyHC genes could hybridise together in the same spots because of their sequence similarity. If this was the case, results would show random patterns in both muscles, but what we have in reality is a particular pattern for *MYH7, MYH1 *and *MYH2 *genes specific to the different muscle types. A larger amount of the *MYH2 *transcript was systematically observed in the PM muscle, which contains less IIA fibres. Different post-transcriptional mechanisms of gene expression, such as siRNA, antisense RNA, RNA interaction with silencing factors, etc., could mediate the relation between *MYH2 *transcripts and IIA fibres.

### Myofibrillar ATPase activity

Muscle studies in cattle have traditionally relied on this method to classify fibre types [15,30,45,46], although in this study the staining for the acid or alkaline stabilities of the mATPase activity was use to determine fibre properties. The immunohistochemistry overcame one of the limitations of the mATPase technique, which could not photometrically distinguish hybrid types with dominance of one isoform from their respective pure phenotypes [11].

Different mATPase profiles have been reported in bovine skeletal muscles. Our results, in agreement with Totland et al. [30], showed that the acid stability of mATPase activity at pH 4.42 was lower for IIA than for IIX fibres (Figures 1E and 3B), whereas the reverse was true for the alkaline stability after preincubation at pH 10.35 (Figures 1F and 3C). However, Picard et al., [3], and Gotoh, [15], found that IIA and IIX (named IIB in their studies) fibres had the same acid stability after preincubation at pH 4.2 and the mATPase stability after alkaline preincubations at pH 10.48 or 10.5 was opposite to ours. This discrepancy was probably related to the different mATPase histochemical methods applied in the different studies, or to slightly different technical procedures. We found significant correlations between mATPase activities and MyHC isoform (Figure 3B–C), which agreed with similar results in a number of other mammalian species [11].

### Metabolic properties

SDH and GPDH activities have been determined in several species, such as goats [39], dogs [11] and swine [18], but, to the best of our knowledge, this is the first study in which they were histochemically quantified in bovine skeletal muscle fibre types. The oxidative and glycolitic capacities differed among fibre types, and showed a negative correlation whose value indicates that the MyHC isoform is not the only factor influencing the metabolic profile of the fibres. This was also reflected in the variation of SDH:GPDH ratios across fibres that, rather, showed quite a remarkable stepwise decline from slow to fast types. The SDH:GPDH ratio expresses the capacity of myofibres for synthesizing ATP from oxidative (SDH) and glycolitic (GPDH) pathways, showing the ability of myofibres to produce energy in aerobic or anaerobic form.

Biological systems have acquired effective adaptive strategies to cope with physiological challenges and to maximise biochemical processes under imposed constraints [19]. Contractile and metabolic properties appeared related in our study (Figure 4D and 4E). Although correlations among MyHC types and metabolic properties were different from zero, the magnitude of such correlations indicated that fibre types did not necessarily exhibit a precise metabolic specialisation. The hybrid fibres had intermediate properties between their respective pure types. However, the metabolic pattern, represented by the SDH and GPDH activities, of hybrid fibres was different to the one described for the contractile properties, in this case indirectly measured by the mATPase activities, as previously described in the Results section (Figures 3A and 4A). In order to assess the effect of both, mATPase and metabolic properties, on the position of hybrid fibres in relation to their pure types, Mahalanobis distances among all groups of fibres were calculated removing mATPase information: the Mahalanobis distances (results not shown) indicated a pattern similar to the one observed in Fig 4a. When metabolic attributes dominated the analysis, hybrid fibres tended to have an intermediate position closer to type IIA in both hybrid fibre populations. However, when the metabolic information was removed, a pattern similar to the one shown in Fig 3A was found. Mahalanobis distances among all groups of fibres were also similar to the ones shown in Table 4. Thus, our results suggest that metabolic and contractile properties appear to position hybrid fibres differently, although they are always between their pure types. Striated muscle tissue demonstrates a remarkable malleability and can adjust its metabolic and contractile makeup in response to alterations in functional demands [19], which could explain the discrepancies between metabolic and contractile patterns.

The fact that the correlation value between the SDH activity and MyHC type was higher than the one between the GPDH and MyHC type indicated that the oxidative specialisation was more preserved among fibre types than the glycolitc one in these muscles and species. Our results, together with previous studies [11,18,39] indicate that the magnitude of the correlations between contractile and metabolic properties differs across species, and such differences could be related to differences in the SDH:GPDH ratio within fibre types among them.

### Histology

The significant differences regarding histological features of the distinct fibre types might have a functional reason, as reported in similar studies [11].

Although the reasons for the difference in the number of nuclei among fibre types are not fully understood, it has been related to different activity patterns among fibre types [47,48]. More active muscle fibres usually have higher levels of both protein synthesis and turnover than those scarcely recruited [11]. Fibres in FD are expected to be more active than fibres in PM, as the number of nuclei were significantly higher in FD than in PM (Table 3). The over-expression of genes related to protein synthesis and turnover observed in FD [42] corroborated this idea.

A small fibre size is an advantage for the diffusion of oxygen and nutrients for oxidative metabolism [49] and is related to more fatigue resistance as well. The mean CSA of the fibre types decreased in the order IIX>IIAX>IIA>I>I+IIA in PM and IIA>I>I+IIA>IIAX in FD, in agreement with a similar study in goats [39] but contrary to a study in dogs, in which the CSA was IIX>I>IIA [11]. An inverse relationship between fibre diameter and oxidative capacity of muscle fibres has been reported [41], which is in accordance with our results.

Capillarisation has been associated with the transport of oxygen and lipids (among other nutrients), and consequently with a large oxidative capacity [41]. In this context, the oxidative capacity of a muscle is related to MyHC isoform distribution, as well as to histological features [40]. Although our study was not designed to compare metabolic activities of the muscles, we observed that all fibre types tended to have a higher oxidative activity in PM than in FD. Therefore, the smaller amount of MyHC I in PM may be compensated for by their large oxidative ability, which could then be more related to capillarisation than to fibre type.

Provided that different motor units are recruited at postural and phasic activities, their constituent muscle fibres might have different sizes and capillary supply [50,51]. Features such as small size and high capillarisation, typical of I and IIA fibre types, mean these motor units are more frequently activated and have a higher oxidative metabolism than the fast IIX motor units. This relationship is also related to the fatigue resistance of the motor units.

Carbohydrates are imported from the capillary supply lines to the myofibres, where they may be stored as either intramuscular triglycerides or glycogen, for later combustion. Fatty acid metabolism is an aerobic process that takes place in the mitochondria [19]. When compared to FD, PM showed a larger relative capillarisation and a smaller cell size, a high expression of mitochondrial genes [42] and a larger IMF content [20]. All these features account for the great oxidative ability of PM in cattle, even when compared to a muscle mainly composed of type I fibres, such as FD.

## Conclusion

Immunohistochemistry allows for the precise identification of three major fibre types containing a single MyHC: I, IIA and IIX, and two hybrid fibre populations, I+IIA and IIAX, in two bovine skeletal muscles (FD and PM). FD was a slow oxidative muscle consisting mainly of I and IIA pure types, which showed a predominant oxidative activity, and without pure IIX fibres. On the other hand, PM was a mixed muscle showing large amounts of I, IIA and IIX pure types, especially IIX. Both muscles had small populations of hybrid fibres, which behaved differently in relation to their corresponding pure types when contractile or metabolic features were considered. To our knowledge, this is the first study in which an accurate and objective classification system is applied to bovine muscles, along with a photometric assessment of relevant contractile, metabolic and histological properties. Coordination between the contractile, metabolic and histological properties of fibres confirmed that the particular expression of a MyHC isoform in a fibre, as well as the quantity of its expression, is related to these properties. The association among them was partial, suggesting that the properties of muscles are not fully explained by variations in the MyHC content.

## Authors' contributions

NMS: muscle sample collection, lab work, image analysis and morphometry, statistical analysis, manuscript preparation. CD: conception, muscle sample collection, statistical analysis, critical revising of the manuscript, substantial contribution to the final manuscript. MJC: statistical analysis, critical revising of the manuscript. JR: manuscript preparation, critical revising of the manuscript. JLR: conception, design, lab work, data collection, statistical analysis, figure preparation, critical revising of the manuscript.
